# Why do people who stutter attend stuttering support groups?

**DOI:** 10.4102/sajcd.v70i1.958

**Published:** 2023-08-03

**Authors:** Nicola E. Bloye, Shabnam S. Abdoola, Casey J. Eslick

**Affiliations:** 1 Department of Speech Language Pathology and Audiology, Faculty of Humanities, University of Pretoria, Pretoria, South Africa; 2 Department of Speech-Language Pathology and Audiology, Faculty of Health Care Sciences, Sefako Makgatho Health Sciences University, Pretoria, South Africa

**Keywords:** dysfluency, people who stutter, perspectives, social support, speech-language therapists (SLTs), stuttering, stuttering support groups (SSGs), quality of life

## Abstract

**Background:**

Stuttering support groups (SSGs) have been a long-standing invaluable resource for people who stutter (PWS) but research into SSGs is only emerging. Speech-language therapists (SLTs) need further insight to successfully facilitate SSGs.

**Objectives:**

To determine PWS’ perspectives regarding why they attend SSGs in South Africa.

**Method:**

Thirteen PWS who attend SSGs, between 20 and 58 years old, were a part of this qualitative study. Purposive sampling was utilised. Semi-structured telephonic interviews were used and data was analysed thematically.

**Results:**

Four themes, namely, ‘altered perceptions’, ‘increased sense of community’, ‘support group reciprocity’, and ‘support group environment, participants and topics’, were identified. The results yielded clinical implications which included SLTs encouraging: (1) improved perceptions of being a PWS through education and self-empowerment, (2) PWS’ connections between meetings to increase the sense of community, (3) reciprocity in meetings, (4) sharing personal stories to promote learning and general self-management and (5) support, praise and education to empower and encourage PWS. This study’s findings show that SSGs helped PWS accept their stutter and gain confidence. This study showcased how SSGs can help PWS manage their fluency and gain confidence. Additionally, this study supports current research which suggests that dysfluency and social-emotional well-being should be equally addressed.

**Conclusion:**

Recommendations were generated from PWS’ perspectives and included focusing discussions on fluency, emotions and sharing personal stories. Insights from PWS helped better inform SLTs of their role within SSGs including guiding and facilitating conversations.

**Contribution:**

People who stutters’ perspectives can be used in clinical practice to help SLTs meet the needs of PWS and guide best practice when facilitating SSGs.

## Introduction

Stuttering is commonly associated with overt disfluent speech; however, the negative emotional and psychological effects have a far greater impact on the lives of people who stutter (PWS). Many PWS experience challenges that extend beyond their ability to communicate, such as high levels of psychological distress, negative affect, fear, shame or embarrassment (Beilby, 2014; Blumgart et al., 2014; Tichenor & Yaruss, 2019; Tran et al., 2011). Social anxiety is also common in PWS and may cause avoidance of speaking situations, potentially leading to feelings of social isolation (Iverach & Rapee, 2014). These difficulties may persist throughout their lives, from educational settings, where negative effects on educational achievement can be seen, to the workplace, where PWS may choose occupations that require less communication (Guitar, 2014; Isaacs, 2021; McAllister et al., 2012; O’Brian et al., 2011). As such, these challenges may result in PWS not reaching their full vocational, occupational and educational potential. The personal and environmental barriers experienced by PWS have been found to limit their participation in everyday activities, with a lower quality of life (QoL) reported in a number of domains (Craig et al., 2009; Figliomeni 2015; Mohammadi et al., 2013; Nang et al., 2018).

Previous research has demonstrated that the QoL of PWS can be improved by attending stuttering support groups (SSGs) (Blumgart et al., 2014; Boyle, 2013). Stuttering support groups are usually facilitated by speech-language therapists (SLTs) and offer a safe environment where PWS can come together as a community and share their feelings and experiences without being judged (Boyle, 2013). Many studies have shown the value of SSGs (Craig et al., 2011; Gerlach et al., 2019; Plexico et al., 2019). Through PWS’ perspectives, past research determined that social support could protect PWS against the negative effects of stuttering and possibly enhance participation in activities of daily living (Gerlach et al., 2019; Tran et al., 2011). Stuttering support groups also play an important role in reducing PWS’ internalised stigma, accepting their stutter and improving PWS’ psychological well-being (Boyle, 2013). Stuttering support groups can provide a setting in which individual stuttering therapy progress can be maintained (Guitar, 2014). It is thus evident that research into the benefits of SSGs is extensive; however, a review of the literature did not showcase any formalised guidelines for SLTs on how to effectively facilitate and manage SSGs.

Although SLTs usually take on the role of facilitating SSGs, it is the community of PWS’ voices that need to be heard to contribute their perspectives and experiences. People who stutter’s perspectives can provide a valuable contribution to the field of stuttering treatment as including people with disabilities in decision-making and goal-setting has been shown to improve clients’ rehabilitation experiences, motivation, and functional outcomes (Brown et al., 2021; Evans, 2012). The degree of motivation can inspire and influence a PWS’ desire to seek intervention or to be productive during therapy (Ratner & Tetnowski, 2014; Sønsterud et al., 2020; Weigel, 2013). It is therefore essential to understand the perspectives of PWS so as to best help them further their personal goals beyond traditional stuttering therapy settings.

People who stutter’s perspectives can provide evidence-based clinical implications that may assist SLTs in tailoring SSG activities, topics of discussion, and goals to better meet the needs of PWS who attend. This research also has the potential to help SLTs to motivate PWS to attend and actively participate in SSG meetings.

## Research methods and design

### Design

The research design was descriptive and phenomenological as the study aimed to understand and describe PWS’ perspectives regarding why they attend SSGs (Leedy et al., 2021; Sandelowski, 2010).

### Setting

A South African SSG served as the research setting. The selected SSG has two groups that run in Gauteng. Both groups meet monthly and are facilitated by SLTs who are experts in the field of dysfluency. At one of the SSG groups, final year SLT students also facilitate, under the supervision of a qualified SLT, and are referred to as ‘SLT students’ in this article.

### Population and sampling strategy

Purposive sampling was used to select PWS who had first-hand experience attending SSGs. The SLTs who coordinate the SSGs were sent a letter requesting permission to recruit members of their respective SSGs to participate. On the researcher’s behalf, the coordinators distributed an information letter and informed consent document to members of their groups. Group members were invited to read the document and ask the researcher questions prior to engaging in the telephonic portion of the research process. Interested members were invited to sign the informed consent document and email it back to the researcher.

The following inclusion and exclusion criteria were applied: (1) be a PWS, (2) be between the ages of 18 and 65, (3) have attended at least three SSG meetings, (4) be able to read and converse in English, (5) have access to an email address and a mobile phone, and (6) have no other self-reported or formally diagnosed communication difficulties. Thirteen people from 20 to 58 years old (mean = 35 years old), three females and 10 males, were selected to participate. Table 1 presents detailed demographic information about the participants.

**TABLE 1:** Participant demographics.

Participant	Age (years)	Age category (years)	Gender	Race	Age of dysfluency onset category (years)	Self-rating of own stuttering severity	Speech therapy history
P1	50	46–55	Male	Indian person	2–6	Mild	Yes
P2	20	18–25	Male	White person	7–13	Moderate	Yes
P3	25	18–25	Male	White person	2–6	Mild to moderate	Yes
P4	27	26–35	Male	Black person	7–13	Moderate	Yes
P5	33	26–35	Male	White person	7–13	Mild	Yes
P6	52	46–55	Female	Coloured person	7–13	Moderate to severe	Yes
P7	34	26–35	Male	Black person	2–6	Moderate	Yes
P8	44	36–45	Female	Coloured person	2–6	Mild	Yes
P9	58	56–65	Male	White person	2–6	Moderate to severe	Yes
P10	26	26–35	Male	Black person	7–13	Mild	Yes
P11	27	26–35	Male	Black person	2–6	Moderate	Yes
P12	30	26–35	Female	Black person	2–6	Moderate	Yes
P13	25	26–35	Male	Black person	2–6	Moderate	Yes

Note: Although the term ‘coloured person’ is now formally recognised as ‘mixed race’, the term ‘coloured’ was the participants’ preferred racial identifier.

The final sample size was determined when information power was reached, as recommended by Malterud et al. (2016). ‘Information power indicates that the more information the sample holds, relevant for the actual study, the lower amount of participants is needed’ (Malterud et al., 2016:1753). The information power was considered to have been reached at 13 participants as the information contained was dense and relevant enough to adequately answer the study’s aim. Data saturation, the point when no additional information is identified, data repeats, and further data collection becomes redundant (Kerr et al., 2010), was also considered and occurred after four interviews.

### Data collection

After providing informed consent, participants were telephonically interviewed in a semi-structured manner for approximately 60 mins. The interview schedule included 34 questions, consisting of both open-ended and closed-ended questions. Closed-ended questions were used to obtain biographic and demographic information. Open-ended questions were used to invite the participants to share information. The questions were divided into four sections: (1) biographic and demographic information, (2) fluency history and behaviours, (3) speech-language therapy treatment, and (4) support groups. The interview schedule was adapted from Medina et al. (2020) with additional questions added to gain further insight into PWS’ perspectives regarding SSGs.

A pre-test was conducted to rule out ambiguous questions and ensure the content and face validity of the interview schedule (Brink et al., 2018; Leedy et al., 2021). After two qualified SLTs reviewed the interview schedule, some questions were altered to ensure clarity and elicit more specific responses from participants. Questions were determined to be representative, clear and appropriate as per the aim of the study.

### Data analysis

Data analysis followed Braun and Clarke’s (2006) six-phase thematic analysis framework. Firstly, recorded interviews were transcribed by the researcher. Secondly, each transcript was re-read for increased data familiarity, and initial codes were generated using ATLAS.ti software (Braun & Clarke, 2006). The initial codes were semantic in nature but were then reviewed to generate latent codes. Maguire and Delahunt (2017) describe semantic codes as those that capture the explicit meaning of participants’ perspectives, while latent codes probe deeper into these perspectives. A bottom-up approach to data analysis was used, as suggested by Terry et al. (2017) where codes were the starting point to develop meaningful themes. As suggested by Braun and Clarke (2006), some of the themes were reviewed twice by the co-authors and all three authors then defined and named the themes. Some descriptive statistics were used to summarise answers to direct (e.g. yes/no) questions.

### Ethical considerations

The Faculty of Humanities’ Research Ethics Committee at the University of Pretoria provided ethical clearance (reference: 17069892 [HUM025/0521]). All participants provided written informed consent and their privacy was maintained by using an alphanumeric code in place of names. Participants’ informed consents, recordings, and transcriptions of their interviews have been securely stored and archived on the University of Pretoria’s data repository.

## Results

During data analysis, four major themes emerged in relation to the SSGs and are as follows: ‘altered perceptions’, ‘increased sense of community’, ‘support group reciprocity’ and ‘support group environment, participants and topics’.

### Theme 1: Altered perceptions

When asked if attending an SSG had a positive influence on their perception of their stutter, 12 participants (92.3%) reported it did alter their perception positively. One participant (7.7%), however, reported no influence. The participant (P5) who experienced no change in their perception of their stutter through attending SSG meetings explained that this was due to negative listener reactions and attitudes; ‘The world out there, still thinks very very bad of us… so I still have a bit of a negative view of not being able to speak fluently’ (P5, 33 year old, male).

#### Subtheme 1.1: Increased acceptance of stutter

Many participants reported SSG meetings helped them to accept their stutter (P1, P3, P4, P6, P8, P9, P10, P11) (*n* = 8; 61.5%). Participants reported that accepting themselves as a PWS played a role in them developing a more positive perception of their stutter (P1, P9, P11) (*n* = 3; 23.1%), influenced their emotional well-being (P4, P8, P10, P11) (*n* = 4; 30.8%) and/or helped them to better cope with their stutter (P3, P10) (*n* = 2; 15.3%). The participants’ quotes can be seen in Box 1.

BOX 1 Quotes for theme 1: Altered perceptions; subtheme 1: Increased acceptance of stutter.

**Table:** 

‘… stuttering … is not the biggest thing in our lives … it’s one part of who we are. It’s something that shouldn’t get the complete focus of our energies.’ (P1, 50 year old, male)
‘… it [*SSG*] provides you with a place to work on that acceptance of your speech …’ (P3, 25 year old, male)
‘… it [*SSG*] helped me a lot … to be able to accept … myself …’ (P4, 27 year old, male)
‘… it’s [*SSG*] also helped me see that it’s not the end of the world.’ (P6, 52 year old, female)
‘I was able to, together with the one-on-one therapy, I was able to accept and embrace the fact that I stutter.’ (P8, 44 year old, female)
‘I realized that my situation is not the end of the world. It could have been a lot worse’. (P9, 58 year old, male)
‘… it’s [*SSG*] taught me to accept the way I am.’ (P10, 26 year old, male)
‘… it [*SSG*] has helped me to really accept that I have a speech problem … and even normalise [*stuttering*] …’ (P11, 27 year old, male)

#### Subtheme 1.2: Improved confidence

Attending SSG meetings helped improve the confidence of some participants (P4, P7, P8, P10, P12) (*n* = 5; 38.5%). Participants reported that their improved confidence allowed them to challenge themselves to complete new tasks and improve their communication. Four participants (P4, P7, P10, P12) (*n* = 4; 30.8%) linked improved confidence to a more positive perception of their stutter. The participants’ quotes are shown in Box 2.

BOX 2 Quotes for theme 1: Altered perceptions; subtheme 2: Improved confidence.

**Table:** 

‘… boosts my self-esteem, to be confident.’ (P4, 27 year old, male)
‘… it [*SSG*] made me more confident speaking to … some people. I’m usually battling with speaking with certain people with their position or status in life … but it [*SSG*] did somehow make me more open …’ (P7, 34 year old, male)
‘In my family, going to the shops, I would get someone else to speak for me because I wouldn’t want to speak. At work, I would get a colleague to ask the boss for something … now I’m the person that does all speaking to everybody else.’ (P8, 44 year old, female)
‘I think again with the confidence, to step out and do things that I wouldn’t normally do.’ (P8, 44 year old, female)
‘… it [*SSG*] gives me more self-control and self-confidence.’ (P10, 26 year old, male)
‘And [*SSG*] also boosts my confidence and I’m free.’ (P12, 30 year old, female)

### Theme 2: Increased sense of community

Participants (P1, P2, P3, P4, P8, P9, P13) (*n* = 7; 53.9%) reported that attending an SSG helped them realise they are not alone in their stuttering journey. These reduced feelings of isolation resulted in PWS (P1, P2, P3, P4, P8) (*n* = 5; 38.5%) having a more positive perception of their stutter, improved emotional well-being (P4, P13) (*n* = 2; 15.4%) and for one participant (P1) (*n* = 1; 7.7%), played a role in coping with their stutter. One participant (P8) (*n* = 1; 7.7%) reported that becoming aware they are not alone improved their speech fluency. Participants’ quotes can be viewed in Box 3.

BOX 3 Quotes for theme 2: Increased sense of community.

**Table:** 

‘… [*the*] support group really helps [*me*] … to not feel alone …’ (P1, 50 year old, male)
‘… [*the SSG*] kind of opened my eyes, that people like me are in the same situation like me …’ (P2, 20 year old, male)
‘… they [*SSGs*] just help you to not feel as isolated…to not feel as if it’s just you and every day you’re going up against your speech and having a tough time on your own …’ (P3, 25 year old, male)
‘I think it helped me a lot … I’m not alone.’ (P4, 27 year old, male)
‘That’s why my fluency increased, because I saw it as it is what it is … I’m not the only one in the world who stutters.’ (P8, 44 year old, female)
‘… it [*SSG*] helped me … to see that you’re not the only one …’ (P9, 58 year old, male)
‘… you know that we’re not alone and there are people out there willing to give you support …’ (P13, 25 year old, male)

### Theme 3: Support group reciprocity

Three participants (23.1%) emphasised the reciprocal nature of SSGs. According to the participants (P1, P2, P5), an SSG is a valuable setting for both sharing and receiving ‘insight’ (P2, 20 year old, male), ‘upliftment’ (P1, 50 year old, male), ‘help’ (P1, 50 year old, male; P5, 33 year old, male) ‘support’ (P1, 50 year old, male), and ‘advice’ (P5, 33 year old, male).

#### Subtheme 3.1: Learning from others

The results in this sub-theme revealed that participants (P1, P2, P4, P5, P9, P12, P13) (*n* = 7; 53.8%) value the opportunity to learn techniques and coping strategies from other PWS. It was mentioned that learning from other PWS was both an effective way to help one cope with their stuttering (P2, P4, P6, P13) (*n* = 4; 30.8%) and an aspect of SSG meetings that participants enjoy (P2, P4, P5, P12) (*n* = 4; 30.8%). Quotes from the participants can be seen in Box 4.

BOX 4 Quotes for theme 3: Support group reciprocity; subtheme 1: Learning from others.

**Table:** 

‘I found that … oftentimes the best way to learn [*is*] from others opinions because you can draw from that.’ (P1, 50 year old, male)
‘… just gaining that insight, and also giving my insights to them. That conversation is what I like the most.’ (P2, 20 year old, male)
‘I … want to learn more and discuss more things.’ (P4, 27 year old, male)
‘I just want to attend these things [*SSG meetings*] just to learn from others, what they did to overcome their problems … It is … good for people who suffer from the same problems to just learn from each other.’ (P5, 33 year old, male)
‘So I definitely think it helped me … to learn a bit from them [*other PWS*] as well.’ (P9, 58 year old, male)
‘I’m learning, you know, each time we have those meetings, there’s always a takeaway point.’ (P12, 30 year old, female)
‘I … get to learn on how other ways of improving my stuttering.’ (P13, 25 year old, male)

#### Subtheme 3.2: Encouragement and empowerment

Five participants (P1, P3, P5, P6, P10) (38.5%) reported that they felt encouraged during and after attending the SSG meetings for a variety of reasons. Participants reported being encouraged and motivated by listening to others’ stories (P1, P10) (*n* = 2; 15.4%) and watching others persevere through difficult disfluent moments (P5) (*n* = 1; 7.7%). P6 explained that praise from other PWS also made them feel more empowered. Lastly, P3 explained that attending an SSG had made them feel more positive and optimistic about their speech. The participants’ quotes are shown in Box 5.

BOX 5 Quotes for theme 3: Support group reciprocity; subtheme 2: Encouragement and empowerment.

**Table:** 

‘… when I come out of a [*SSG*] meeting, I always feel inspired … because I’ve just come from a platform where people have been honest, really, I mean people have stripped themselves, to bare, to expose themselves as much as they did to, to explain the core feelings which they actually experiencing …’ (P1, 50 year old, male)
‘It’s encouraging. You feel as if there’s like people who are trying to help. You’re not fighting the battle on your own …’ (P3, 25 year old, male)
‘It [*SSG*] definitely does help you to feel a bit more positive and optimistic about your speech.’ (P3, 25 year old, male)
‘… it [*SSG*] does give me a bit of hope because, for example, at the very first [*SSG meeting*], there was a person who struggled to speak way more than what I ever did, and he did overcome it, um, so it was quite inspiring for me to see …’ (P5, 33 year old, male)
‘… after you speak, then somebody would like say something or, “well done”, or “that was good”, so it’s very encouraging …’ (P6, 52 year old, female)
‘… when you walk away from there, you feel a little bit empowered and it’s going to be okay, the team encourages you …’ (P6, 52 year old, female)
‘The things that they [*other PWS*] were saying, it motivates me …’ (P10, 26 year old, male)

### Theme 4: Support group environment, participants and topics

#### Subtheme 4.1: Environment

Participants (P1, P2, P3, P4, P6, P7, P8, P10, P12) (*n* = 9; 69.2%) expressed how the SSG meetings created an environment where they felt heard, safe, relaxed, free and/or experienced a sense of belonging. Some of the participants’ (*n* = 5; 38.5%) quotes can be seen in Box 6. Some participants (P4, P5, P13) (*n* = 3; 23.1%) voiced their desire for monthly SSG meetings to occur more frequently, with a wider range of dates and times for greater flexibility.

BOX 6 Quotes for theme 4: Support group environment, participants and topics; subtheme 1: Environment.

**Table:** 

‘I’ve got this off my chest and at least someone’s heard me. I’ve been heard.’ (P1, 50 year old, male)
‘… they’re [*other PWS*] not going to judge, or things like that. It’s a place to speak openly about things like that.’ (P2, 20 year old, male)
‘The atmosphere of the meeting … really plays a big role, it’s not a serious conversation, it’s … an open heart-to-heart conversation …’ (P2, 20 year old, male)
‘… [*the SSG*] … has been an important safe space where you can express your emotions and … [*discuss*] the emotional aspects of disfluent moments … that really helps a lot.’ (P2, 20 year old, male)
‘[*The SSG*] to me is a support environment …’ (P3, 25 year old, male)
‘I feel more relaxed …’ (P4, 27 year old, male)
‘What I enjoy about [*the SSG*] … is … we don’t judge.’ (P4, 27 year old, male)
‘Nobody laughs at you … it’s a safe place.’ (P6, 52 year old, female)
‘It’s an open environment.’ (P7, 34 year old, male)
‘It [*SSG*] was the one platform [*where*] you can speak, and no one gives a rat’s ass.’ (P8, 44 year old, female)
‘… feel [*though*] we are home.’ (P10, 26 year old, male)
‘I always feel as if I’m different and so when I’m at [*the SSG*], I’m at home and … I feel like I belong.’ (P12, 30 year old, female)
‘Sometimes the time is not convenient as such.’ (P4, 27 year old, male)
‘… maybe like two options [*of dates for SSG meetings*] per month that people can choose one of the two, that would be good.’ (P5, 33 year old, male)
‘I wish that maybe … we could have more sessions …’ (P13, 25 year, male)

#### Subtheme 4.2: Value of speech-language therapists as facilitators

Two participants (P3, P6) (*n* = 2; 15.4%) expressed that they enjoyed having SLTs and SLT students as SSG facilitators. One of these participants mentioned that having SLTs at the meetings allowed those who stutter and those who treat stuttering to collaborate – ‘it’s so valuable as well to have … that collaboration between like the people who experience it every day and then the people who are actually educated on it’ (P3, 25 year old, male). P3 also felt encouraged that SLTs were trying to help and advocate for PWS – ‘It’s encouraging. You feel as if there’s like people who are trying to help’ (P3, 25 year old, male). The other participant, P6 (52 year old, female) (*n* = 1; 7.7%), stated that they believed SLTs should participate to ‘guide the conversation,’ ‘coordinate it [*an SSG meeting*] with professionalism’, and that SSG attendees are ‘aligning to the agenda’. P6 also expressed that the unique theoretical and clinical knowledge SLTs bring to SSG meetings further reinforced their value. P6 explains, ‘they’ve [*SLTs*] got the knowledge that’s different to us living with it [*stuttering*]. So I understand myself, but they would understand everybody’ (P6, 52 year old, female). P2 (7.7%) mentioned that:

‘there were times when [*they were*] the only participant in the group that was a stutterer and it felt like all of the attention was on [*them*], and almost like an interrogation.’ (P2, 20 year old, male)

P2 therefore suggested that SLTs should also answer questions and share their insights to prevent PWS feeling as though they are being interrogated.

#### Subtheme 4.3: Meeting topics

Participants (P2, P3, P6, P7, P8) (*n* = 5; 38.5%) suggested SSG meetings should focus on speech and dysfluency. These participants suggested that topics should involve ‘… things going on in the world of dysfluency …’ (P3, 25 year old, male) and be ‘centred around stuttering’ (P6, 52 year old, female). P7 (34 year old, male) suggested that activities be ‘… more stuttering related’. P8 (44 year old, female) reported that ‘unpacking all the emotions attached to stuttering’ during SSG meetings was beneficial. P3 expressed that they found it positive when SSG meetings were ‘educational but … supportive at the same time’ (25 year old, male). P2 said:

‘… [*the SSG*] has been an important safe space where you can express your emotions and … the emotional aspects of disfluent moments and sharing that, talking about it, that really helps a lot …’ (P2, 20 year old, male)

## Discussion

People who stutter and attend SSGs shared their thoughts and opinions on why they attend SSGs. Their perspectives highlighted important clinical implications for SLTs who facilitate SSGs. These clinical implications were used to inform recommendations for SLTs, such as guidance on the role of SLTs in meetings, the purpose and structure of SSGs, and suggested topics of discussion and activities.

### Altered perceptions

As one participant reported, and in congruence with prior research, negative listener reactions can negatively impact PWS’ perception of their stutter (Bajaj et al., 2017; Yaruss & Quesal, 2004). It is therefore important that SLTs facilitate interactions that include relatives and listeners who are not PWS. During SSGs, SLTs can provide PWS with means to encourage positive interactions, such as sharing individual experiences, or using self-advertising or self-disclosure statements, which have been reported to yield more positive listener reactions (Kittilstved, 2014; McGill et al., 2018).

The findings identified that SSGs aided PWS in accepting their stuttering, which contrasts with previous research completed by De Nardo et al. (2016) who found no link between support groups and self-acceptance. One participant reported that stuttering is part of who they are and should not define their individual identity, raising the idea of stuttering and identity. Similarly, Blumgart et al. (2014) and Boyle (2013) found that attending SSG meetings can result in a changed self-identity as a PWS, and improved self-acceptance. As self-acceptance of a PWS is linked to an improved QoL (Swartz et al., 2014), SLTs should address acceptance during SSG meetings. According to Sheehan (2018), education promotes self-acceptance. Speech-language therapists can ensure that PWS receive a holistic, comprehensive, and accurate understanding of their stuttering, possibly through presentations, guest speakers, and question-and-answer sessions. Speech-language therapists can also encourage self-empowerment by using ‘I’ statements when referring to stuttering, and validate members’ stories, vulnerable moments and honesty, to foster an accepting environment, and ensure that no member’s efforts to share are dismissed (Sheehan, 2018).

People who stutter reported increased participation in previously avoided tasks as a result of increased confidence, a finding that is supported by Blumgart et al. (2014). Gore and Luckman Margulis (2022) proposed activities that can improve confidence in a therapy setting. These activities can be adapted for SSGs and can include sharing stories about successful communication interactions, discussing ways to foster cognitive resilience and combat negative reactions. Speech-language therapists should emphasise that SSGs encourage a sense of hope and agency for future communication opportunities.

### Increased sense of community

A supportive social network fosters greater psychological resilience (Boyle, 2015) which can protect PWS from the negative psychosocial effects of stuttering including social isolation (Craig et al., 2011; Gerlach et al., 2019; Iverach & Rapee, 2014). One PWS stated they had previously formed a buddy system with another PWS in their SSG where they would communicate with each other and practise fluency techniques between SSG meetings. Speech-language therapists can facilitate a supportive network by creating a secure platform where members who wish to connect outside the SSG setting can share their contact details. These connections, which extend beyond a pre-arranged group meeting, may further increase the sense of community and reduce feelings of isolation. The expressed desire for increased frequency of SSG meetings indicates PWS’ interest in more frequent connection and can increase the sense of community within the SSG. Speech-language therapists could also use a hybrid approach for SSG meetings, where both in-person and online meetings are available. A hybrid approach removes a location barrier, may improve attendance and can increase the number of SSG attendees. Although research has shown that rehabilitation services can be made further accessible through an online platform (Molini-Avejonas et al., 2015), it is still important to consider that many people in South Africa do not have access to the means needed for online services. Further research could therefore investigate how SLTs can make SSGs more accessible for PWS throughout South Africa.

### Support group reciprocity

‘Learning from others’ and ‘encouragement and empowerment’ were the two subthemes under theme three. Under each of these subthemes, participants alluded to the theme of reciprocity of an SSG.

Participants expressed value in learning coping techniques from other PWS because it gave them insight from peers who could relate to them and their experiences. Past research has shown that listening to a PWS share their own story can reduce the stigma associated with stuttering (Boyle et al., 2016). Facilitating SLTs could give PWS the opportunity to share their stories and encourage active listening to help reduce the negative stigma associated with stuttering. Speech-language therapists could also invite guest speakers who stutter or who are professionals in the management of stuttering. Group meetings can be an ideal setting for people with disabilities to learn about self-management, which can be facilitated by members sharing their knowledge and skills with others (World Health Organization [WHO], 2010).

Thoits’ (2011) discussion found that SSGs allow PWS to share their feelings and worries, receive support and affirmation, and be compassionately understood. Similarly, the participants in this study wanted to support and be supported by others and to uplift and be uplifted. Participants also felt encouraged by watching others with greater stuttering severity persevere through difficult stuttering moments. Future research should investigate the impact of PWS’ perception of their stutter when they meet PWS with less severe stuttering than their own. People who stutter and SLTs need to be informed about how this experience may impact a person’s reaction in order to address this within SSGs. Receiving support and praise from other PWS was both encouraging and empowering for the participants in this study, a finding that is also supported by previous research (Tichenor & Yaruss, 2019).

As the combination of education and empowerment is shown to effect positive change, empowerment is an important component of stuttering therapy (Gore & Luckman Margulis, 2022). Empowerment has also been found to be achieved through gaining knowledge and information (Barak et al., 2008). Speech-language therapists who facilitate SSGs can help PWS learn about their stuttering from other PWS and professionals. Speech-language therapists can encourage group members to share stories about self-disclosing their stuttering, and personal tips (Gore & Luckman Margulis, 2022). An SSG can also provide a safe environment in which PWS can apply and troubleshoot self-disclosure techniques taught in individual speech therapy. Speech-language therapists could encourage members to discuss any stigma they have encountered in their lives and share strategies they used to challenge or overcome this stigma (Gore & Luckman Margulis, 2022). Speech-language therapists can also encourage friends and family members to attend meetings occasionally, and centre the agenda on information about stuttering and ways in which they, as family members and friends, can best help PWS.

### Support group environment, participants and topics

According to Craig et al. (2011), social support promotes a sense of belonging. Similarly, the PWS in this study valued how the SSG’s supportive environment made them feel heard, safe, and relaxed, and/or gave them a sense of belonging. Therefore, to help all members feel heard, SLTs could monitor each member’s contribution in the SSG meeting, and direct questions or points of discussion to those who have not contributed as much. People who stutter did, however, value knowing that they are not expected to speak during the SSG setting, should they not wish to. Therefore, SLTs should reassure members that they are not required to contribute verbally in order to attend SSG meetings. Maintaining confidentiality is also important in the field of SSGs as it has been linked to increased self-disclosure within a group setting (Doshi et al., 2019). The SLT can request that members do not share personal information, details and/or stories about their fellow group members with others outside of the SSG. The WHO, however, highlights that different cultures may view confidentiality in various ways and therefore suggests that the rules of confidentiality be decided by the group (WHO, 2010).

Participants expressed the opinion that SSG meetings should focus on speech and dysfluency, in addition to addressing the emotional aspects of stuttering. As the SLT is often the person who creates the agenda for the meeting, he or she must ensure both of these topics are covered. To assist with this, SLTs could ask members at the end of each SSG meeting what topics they would like covered in the next meeting. The researcher was unable to locate any studies that have been conducted to date, with the purpose of determining what topics PWS would like to discuss within SSG meetings. This is therefore a topic that could be researched further in order to ensure that the topics discussed are of interest, importance, and relevance to the SSG members to best meet their needs.

The WHO proposed that people with similar disabilities, as well as rehabilitation professionals should share information, ideas, and experiences to encourage mutual understanding and collaboration (WHO, 2010). This is supported by this study as PWS value and enjoy having SLTs facilitate support groups. In previous research, people who stutter expressed appreciation for the facilitators of their SSG meetings. They specifically valued how facilitators ensure equal speaking opportunities for members, allowed members freedom to go off-topic, generated topics for meetings and planned the meetings (Medina et al., 2020). People who stutter in this study support and further expand on findings by Medina et al. (2020) regarding their perspectives of the SLTs’ role within the SSG. People who stutter in this study suggested that SLTs collaborate as much as possible with PWS during SSG meetings, advocate for PWS, facilitate conversations in which SLTs can develop a deeper understanding of PWS and their experiences, and share their clinical and theoretical knowledge. Although the PWS in this study viewed SLTs as a positive presence in their respective support groups, past research has shown that SLTs’ presence can be perceived negatively if they are only there for observational purposes (Trichon, 2007). Therefore, SLTs must strike a balance between active and passive participation.

## Conclusion

This study used the perspectives and voices of PWS, along with previous research, to guide evidence-based recommendations for SLTs who facilitate SSGs. The PWS in this study expressed that they valued having a welcoming, safe, and relaxed environment. Findings also highlighted that SLTs need to strike a balance between being passive and active participation, so that they do not over-contribute but are also not perceived as observers. Topics for SSGs could include fluency as well as the emotional aspects of stuttering. Speech-language therapists can use SSGs to help PWS have a more positive attitude towards themselves and their stuttering, as well as provide the tools and resources they need to have more positive experiences when speaking with people who do not stutter. Interestingly, PWS suggested that forming relationships outside of the SSG should also be encouraged. Speech-language therapists can also encourage SSG members to share their stories when they are ready, and facilitate the discussion so that all members benefit from each meeting. As suggested by PWS, facilitating SLTs should also consider increasing the frequency of meetings. Speech-language therapists can empower members by educating them about their stuttering and facilitating conversations in which PWS can learn from other PWS and professionals.

With these clinical implications derived from PWS’ personal perspectives and opinions, facilitating SLTs may now be able to better tailor activities, topics of discussion, and goals, for the PWS who attend SSGs. This may encourage PWS to pursue personal goals outside of traditional stuttering therapy settings and experience a greater sense of confidence across a variety of communication settings.
