# Erratum: Why do people who stutter attend stuttering support groups?

**DOI:** 10.4102/sajcd.v71i1.1046

**Published:** 2024-03-22

**Authors:** Nicola E. Bloye, Shabnam S. Abdoola, Casey J. Eslick

**Affiliations:** 1 Department of Speech Language Pathology and Audiology, Faculty of Humanities, University of Pretoria, Pretoria, South Africa; 2 Department of Speech-Language Pathology and Audiology, Faculty of Health Care Sciences, Sefako Makgatho Health Sciences University, Pretoria, South Africa

In the published article, Bloye, N.E., Abdoola, S.S., & Eslick, C.J. (2023). Why do people who stutter attend stuttering support groups? *South African Journal of Communication Disorders, 70*(1), 958., there was an error with the second authors affiliation.

Instead of:

^2^Department of Speech-Language Pathology and Audiology, Faculty of Health Care Sciences, Sefako Makgatho Health Sciences University, Pretoria, South Africa

It should be:

^1^Department of Speech Language Pathology and Audiology, Faculty of Humanities, University of Pretoria, Pretoria, South Africa

The publisher apologises for this error. The correction does not change the study’s findings of significance or overall interpretation of the study’s results or the scientific conclusions of the article in any way.

